# HapticWhirl, a Flywheel-Gimbal Handheld Haptic Controller for Exploring Multimodal Haptic Feedback

**DOI:** 10.3390/s24030935

**Published:** 2024-01-31

**Authors:** Jose Luis Berna Moya, Anke van Oosterhout, Mark T. Marshall, Diego Martinez Plasencia

**Affiliations:** 1 Creative Technology Group, Department of Informatics, University of Sussex, Brighton BN1 9PH, UK; j.berna@sussex.ac.uk; 2 Industrial Design, Eindhoven University of Technology, 5612 AE Eindhoven, The Netherlands; a.v.oosterhout@tue.nl; 3 Interaction Design Centre, University of Limerick, V94 T9PX Limerick, Ireland; 4 Department of Computer Science, University College of London, London WC1E 6BT, UK; d.plasencia@ucl.ac.uk

**Keywords:** VR, ungrounded force feedback, gyroscopic effect, flywheel, controller design

## Abstract

Most haptic actuators available on the market today can generate only a single modality of stimuli. This ultimately limits the capacity of a kinaesthetic haptic controller to deliver more expressive feedback, requiring a haptic controller to integrate multiple actuators to generate complex haptic stimuli, with a corresponding complexity of construction and control. To address this, we designed a haptic controller to deliver several modalities of kinaesthetic haptic feedback using a single actuator: a flywheel, the orientation of which is controlled by two gimbals capable of rotating over 360 degrees, in combination with a flywheel brake. This enables the controller to generate multiple haptic feedback modalities, such as torque feedback, impact simulation, low-frequency high-amplitude vibrations, inertial effects (the sensation of momentum), and complex haptic output effects such as the experience of vortex-like forces (whirl effects). By combining these diverse haptic effects, the controller enriches the haptic dimension of VR environments. This paper presents the device’s design, implementation, and characterization, and proposes potential applications for future work.

## 1. Introduction

VR technology has seen significant advancements, transitioning towards lightweight, portable wearable headsets that offer immersive virtual environments at high resolutions and refresh rates. Despite the pivotal role of haptic feedback in how we experience a Virtual Environment (VE), haptic controllers have not yet experienced such a leap. This is partly due to the fact that while great progress has been made beyond using eccentric rotating mass vibrators that generate an on-off stimulus, most actuators can only generate a single haptic modality, thereby limiting the depth of expressiveness that they can generate.

Kinaesthetic haptics presents significant challenges, and its success in the realm of commercial consumer devices has been relatively limited [1,2]. This limitation can be attributed to the prevalent trend of designing such haptic devices with specific use cases in mind, resulting in a lack of broader applicability. Particularly in the context of hand-held interfaces, which are most naturally suited for untethered VR experiences, the challenges become more pronounced.

In the realm of kinaesthetic haptics, a common limitation prevails across various haptic devices—the capacity to produce only a single haptic modality. For instance, controllers like CLAW [3] and CapstanCrunch [4] focus on delivering controllable stiffness during pinching interactions, while the Thor’s Hammer [5] and Aero-plane [6] handheld haptic controllers employ propellers to generate forces and torques. Furthermore, active force feedback exogloves such as CyberGrasp [7] and Dexmo [8] constrain the user’s movements in order to replicate collisions and interactions by using mechanical links or Bowden cables. Hand-held devices with indirect actuation, such as Shifty [9], Transcalibur [10], and Drag:on [11], provide haptic interactions based on the controller’s inertia, but they remain confined to specific modalities. These examples underscore the prevailing limitation of existing haptic controllers, wherein they can only produce a singular type of haptic stimulus, thus restricting the breadth of haptic experiences they can offer.

In this paper, we introduce an innovative haptic controller: an untethered handheld device that leverages a flywheel-based system capable of delivering diverse haptic effects. These encompass torque feedback, impact simulation, low-frequency, high-amplitude vibrations, the sensation of momentum (inertial effects), and complex haptic output effects such as the experience of vortex-like forces (whirl effects). The flywheel, actuated using two 360-degree gimbals, can orient in any direction at various speeds to generate these effects. Additionally, the flywheel is equipped with a clamp brake that allows for sudden stopping of the flywheel.

The uniqueness of our proposed solution lies in its ability to deliver a varied output of haptic feedback modalities using a single actuator. This paper provides a comprehensive account of the device’s design, implementation, and characterization, as well as a discussion of its potential future applications. This serves as the groundwork for future work to further explore and develop opportunities with this type of actuator. We explore the device’s integration within virtual environments and delve deeper into its potential use cases in the simulation of forces, recreating vibrations, and impact effects. Measurements of the controller output torque, vibrations created using a brake to stop the flywheel, and vibrations created using the inner flywheel are all captured to validate the effectiveness of our proposed solution. Our device is also open source and open hardware, allowing it to be built by anyone with an interest in experimenting with haptic interfaces and opening up possibilities for experimenting with multiple haptic effects when interacting with virtual reality environments (see [12] for more details).

In the subsequent sections, we delve deeper into the device’s design and characterization. In addressing the limitations of existing haptic technologies, this paper introduces a unique, untethered VR device utilizing a flywheel-based system for multi-modal haptic feedback. Our significant contributions are as follows:

- We present a design and implementation approach for a flywheel actuator to be integrated on a handheld haptic controller capable of delivering diverse haptic effects using only this single actuator.
- We provide an in-depth analysis of the operation and control of the flywheel and gimbal combination to enable further development of the device.
- We offer a comprehensive characterization of the proposed device, backed by extensive measurements of controller output torque and vibrations. This empirical analysis demonstrates the effectiveness of our proposed solution.
- We describe potential applications of this device, showing how it can enrich the tactile dimension of VR environments.

## 2. Related Work

In this section, we discuss related work on gyroscope feedback devices and force feedback implementations for VR.

### 2.1. Torque Gyroscope Feedback Devices

Inertia can be generated by the angular momentum of a flywheel spinning at a high speed. Changes in speed on the flywheel or changing the rotation axis orientation create an output force orthogonal to the flywheel plane. The direction of the output torque depends on the rotational direction of the wheel and its re-orientation angle and speed. The device developed by Yano et al. provides two degrees of freedom and has been evaluated for the detection of objects, guidance, and an enhanced user’s moment [13]. More recent work has explored different applications of the gyro effect, showing how the output torque can induce a wrist twist while swinging the arm [14].

Altering the speed of the flywheel to create and output torque around the axis of rotation has been explored as a means of delivering directional cues. GyroCube [15,16] presents a method that uses three individual flywheels arranged in a hand-held cube. Directional force feedback could be experienced by sequentially switching on one actuator at a time. Some studies explored adding a mechanical brake to the flywheel to generate directional force feedback on wearable devices to provide directional guidance [17,18]. Other examples use multiple gyros to counteract undesired torque effects when returning the gyro to the home position [19,20]. The results of the study by Amemiya et al. [17] suggest that an angular velocity change with a sudden-start profile is more effective compared to sudden-stops.

In the context of mobile applications, TorqueScreen [21] and GyroTab [22] provide force feedback for tablet devices. GyroTab uses a flywheel to provide torque feedback reactive to the user’s movements, while the flywheel that is added to TorqueScreen can impose angular momentum on the tablet without user movement by rotating over one axis. A handheld controller developed by Winfree et al. [23] integrated the flywheel in a two-degree-of-freedom cage similar to the design from Yano et al. [13], but with further improved control design. They also provided a thorough evaluation of the torque that can be generated with the device and in-depth operation. Building on their work, we explore how such forces can be applied in VR, and evaluate the torque generated by our device and the impact of the mechanical brake.

There are also some gyroscope designs that directly focus on VR interactions, such as GyroVR [24], which explored the use of head- and body-worn flywheels to support motions like flying, diving, or floating. Recent work explored how the feedback created by a flywheel can be used to correct the posture of the golf club while swinging it using a single pivot axis point for the flywheel [25].

However, these devices typically employ the gyroscopic effect in a singular modality. Our work with HapticWhirl diverges by exploring multiple modalities through the control of the flywheel and its orientation. This approach enables a broader spectrum of haptic experiences in VR, ranging from inertia simulation to directional feedback, thereby expanding the potential applications and user experiences within virtual environments.

### 2.2. Haptic Interfaces for VR

#### 2.2.1. Motor-Actuated Devices

For immersion in virtual environments, force feedback has been implemented in many ways to mimic different kinaesthetic experiences. Head-mounted attachments like FacePush [26], which uses servo motors and heights attached to the side of the VR headset, can generate push and pull forces. Thor’s Hammer [5] uses propellers to induce ungrounded force feedback in a handheld haptic device. It combines six propellers to generate force feedback in three dimensions. With the strong and continuous force feedback that can be generated with the device, interactions like moving an object through water or simulating the weight of an object can be supported. The light weight of the propellers makes them more suitable for VR applications and wearables when compared to flywheels. The wind blaster [27] is an example of a wrist-worn device that provides force feedback using two propellers. PaCaPa [28] offers a simple mechanical handheld device to make indirect interaction with handheld tools in VR more realistic.

#### 2.2.2. Weight Shifting

Several studies have utilized the concept of weight shifting to generate haptic experiences for VR. SWISH [29] is able to reproduce the physical sensation of fluid vessels. The implementation uses a mechanical construction to change the horizontal and vertical positions of a mass in the cylinder. Shifty [9] controls only the vertical position of a weight proxy to generate weight-shifting feedback for handheld devices. Cheng et al. [30] have taken a different approach and used liquid-based haptic feedback to simulate a realistic weight for virtual objects, using pumps and water tanks. While Drag:on [11] adjusts its surface area to vary the resistance when the controller is moved, the effect can be used to simulate the weight of the objects moved by the user in VR. In addition to weight simulation, Transcalibur [10] alters its shape and center of mass to mimic the haptic feel of different weapon-type controllers.

#### 2.2.3. Elastic Interfaces

Force feedback can also be generated by varying the tension of elastic bands. ElasticVR [31] and ElastImpact [32] are wearable devices that can generate impact forces instantly by releasing power that was stored in elastic bands in advance. The same principle is applied in ElastOscillation [33], a device that can provide a damped oscillation effect at 3 degrees of freedom. Similarly, Elastic-Arm [34] uses an elastic body attachment between the shoulder and the user’s hand to provide passive feedback.

#### 2.2.4. Exogloves

An alternative untethered design for limb force feedback is making use of the user body as an anchor point for actuators. Cable-driven designs such as Wireality [35] use a spring-loaded cable system mounted on the shoulder. The cables attach to multiple joints on the user’s hand, allowing for locking action when a collision on the VE is detected. Some exosuits [36,37] use a similar cable/clutch design, integrating the bowden cables on the suit or soft fabric and creating similar motion constraints via passive actuation. Wearables with more advanced control designs [38] include brushless motor actuators attached to the body attachment, allowing for more realistic force feedback and active effects. The controller presented in this paper aims to provide feedback for continuous motion as well as impact by combining a flywheel that can rotate in 2 degrees of freedom and a brake mechanism.

### 2.3. Multimodal Kinaesthetic Feebdack

Handheld devices for virtual reality, while providing a broad workspace and ease of use, typically only offer single-mode haptic feedback, such as vibration [39]. However, to truly enhance the user’s immersive experience, multimodal haptic feedback is required, often necessitating the use of various types of actuators to simulate different tactile submodalities like force, shape, texture, shear, or temperature.

For instance, NormalTouch and TextureTouch devices, developed by Benko et al. [40], provide force feedback and simulate the shape and texture of virtual objects. These devices use a movable platform and an array of actuated pins to create an experience of interacting with 3D shapes.

Choi et al. [41] designed a device that combines a gripping mechanism with asymmetric skin stretch to simulate touch, grasp, gravity, and inertia. It creates a sense of holding a weighty object and offers a wide range of motion. A unidirectional brake and two voice coil actuators provide a perception of gravitational and inertial forces.

The claw controller [3], another innovation by Choi et al., is a multipurpose controller providing haptic feedback to the index finger. It can simulate the sensation of various textures, holding soft or rigid objects, and triggering a gun.

Whitmire et al. proposed the Haptic Revolver [42], a device capable of simulating touch contact, pressure, shear forces, textures, and shapes. This is accomplished through a rotating wheel beneath the user’s index finger, which rises and falls to imitate contact with virtual surfaces.

Tasbi [43] is a multimodal haptic wristband developed by Pezent et al. that provides radial squeeze forces around the wrist along with vibrotactile feedback at six discrete locations around the band. The device uses a DC motor to drive the squeezing mechanism, which minimizes tangential forces between the band’s points of contact with the skin. Force-sensing capacitors enable closed-loop control of the squeeze force, while linear resonant actuators provide vibrotactile feedback.

In summary, while many handheld devices offer a single perceptual haptic submodality, to achieve a more complete, immersive user experience, designers incorporate different types of actuators to generate multimodal haptic feedback.

## 3. Controller Design

In this section, we discuss the design of the HapticWhirl controller, including its hardware implementation, working principle, and haptic control methods. This manuscript details the development of a haptic device, termed HapticWhirl, which employs a flywheel actuator to generate multimodal 3-DOF force feedback. The device is constructed using materials and components readily accessible and manufactured through FDM 3D printing.

### 3.1. Hardware Implementation

The HapticWhirl main actuator is a brushless motor and flywheel disk repurposed from a conventional hard disk drive. The motor’s flat package design facilitates its alignment with the gimbal’s center or mass. The total weight of the rotating disk and motor’s rotating body is 96 g, with a diameter of 95 mm for the disk (flywheel). The motor speed is controlled by a generic 30A electronic speed controller (ESC), which allows it to reach rotational speeds of up to 12,000 RPM.

**Figure 1:**
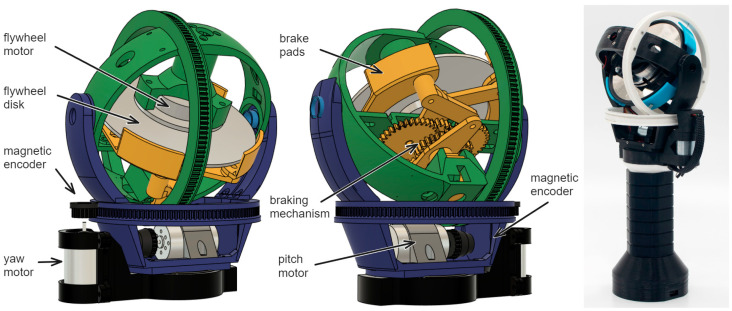
The physical components of the HapticWhirl controller. Blue is used to highlight the components that are part of the Z axis gimbal, green for the Y axis and yellow for all the components of the braking mechanism.

The fundamental mechanism for generating torque with the flywheel involves altering its orientation while the flywheel is rotating. For this purpose, the disk/motor assembly is mounted on a mechanically actuated spherical gimbal frame, providing two rotational degrees. When the HapticWhirl controller is held vertically and in the rest/home position, the disk remains horizontally oriented. Gimbal mechanisms are powered by Maxwell 110147 DC motors (Maxon Group, Wokingham, UK) through a pulley system, achieving gear ratios of 10:1 for yaw rotation and 11.5:1 for pitch, amplifying the motor’s output torque while maintaining a rotational speed of up to ~10.45 rad. A compact Printed Circuit Board (PCB) (JLCPCB, Guangdong, China) housing a 12-bit resolution digital encoder (AS5047, ams-Osram AG, Premstaetten, Austria) is positioned opposite the capstan pulley, which is equipped with a magnet. The direction and speed of the gimbal motors are controlled using an H-bridge controller (LN298, ST Microelectronics, Crolles, France). The gimbals are capable of rotating over 360 degrees; for this, slip rings are installed on both axes. The motors for each axis utilize 3D-printed Miniature Extra Light (MXL) belts, which we printed in-house, made from Ninjaflex 95A material and MXL gears printed using PLA. This design allows continuous rotation speeds of 8.35 rad/s (~800 rpm). See Figure 1.

To enhance the haptic feedback of HapticWhirl, the design includes a scisor drum brake mechanism on one side of the gimbal, enabling rapid stopping of the flywheel. This mechanism, actuated by an SG90 microservo, can halt the flywheel in 0.8 s. Full details and links to download all the 3D parts and designs are freely available [12].

In the final design, the gimbal has an outer diameter of 125 mm, and the controller’s total height, from the bottom of the handle to the top, is 260 mm. The total weight of the controller, including an HTC Vive tracker that is mounted at the base for 3D tracking, is 720 g.

**Figure 2:**
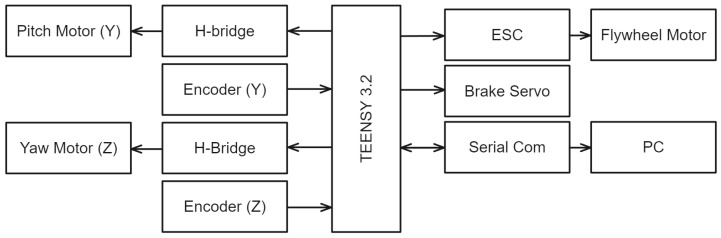
The controller’s inputs and outputs. Left components represent the gimbal control sensors and actuators, on right flywheel components and communication.

The flywheel motor and gimbal motors operate on a 12 V supply, whereas the other electronics function at 3.3 V. A Teensy 3.2 microcontroller executes a control loop at 500 Hz and communicates with the PC via serial communication at a baud rate of 115,200. Real-time data from the encoders is used to determine the orientation and angular speed of the flywheel, integral to the closed-loop control system. See Figure 2.

### 3.2. Working Principle

The HapticWhirl device generates two primary kinaesthetic forces, originating from its flywheel and the dual actuated gimbals. This device leverages the gyroscopic effect by adjusting the orientation of the spinning flywheel through these gimbals. As the flywheel rotates, its orientation changes and it encounters resistance, perceived as torque ($\vec{\tau }$) acting perpendicular to the rotation plane. This torque elicits a tactile sensation of push or pull for the user. The gyroscopic effect’s operation in the HapticWhirl can be quantified with a set of equations. The torque generated by the flywheel is the rotational force arising from the flywheel’s change in angular momentum ($\vec{L}$). The output torque is a combination of the flywheel’s angular momentum:

(1) $$\vec{\tau }=\frac{d\vec{L}}{dt}=I\frac{d}{dt}{\vec{\omega }}^{\mathrm{disk}}=I{\dot{\vec{\omega }}}^{\mathrm{disk}}$$

In this equation, the torque ($\vec{\tau }$) is a product of the flywheel’s moment of inertia (I) and the rate of change of its angular velocity (${\dot{\vec{\omega }}}^{\mathrm{disk}}$). This is the fundamental torque generated by rapidly altering the speed of the spinning flywheel using the brake mechanism. When the flywheel’s speed changes abruptly, a reactive torque manifests according to the law of conservation of angular momentum, acting in the direction opposite to the disk’s rotation during acceleration or in the same direction during deceleration using the drum brake. Building on this, the steered momentum wheel concept is introduced in the equation:

(2) $$\vec{\tau }=\frac{d\vec{L}}{dt}={\vec{\omega }}^{gimbal}\times I{\vec{\omega }}^{disk}$$

represents the dynamics of the gimbal, changing the orientation of the flywheel. Here, the torque ($\vec{\tau }$) is a product of the interaction between the gimbal’s angular velocity (${\vec{\omega }}^{gimbal}$) and the flywheel’s angular momentum (${\vec{L}}^{flywheel}$). This interaction explains the additional momentum generated when both components move simultaneously. The combination of both equations describes the overall system dynamics within frame of reference A (origin at the center of the flywheel):

(3) $${\vec{M}}_{A}^{gyro}=I\cdot {\dot{\vec{\omega }}}_{A}^{Disk}+{\vec{\omega }}_{A}^{Gimbal}\times (I\cdot {\vec{\omega }}_{A}^{Disk})$$

Each one of these components can be expanded, combined, and simplified to present the operation of the controller with a single equation for each of the 3D components, as a factor of pitch (ω), yaw (ψ) and disk angle (ρ), full step by step details of the evaluation of the mathematical model are available online with schematics and visual aids [12]:

(4) $${\vec{M}}_{A}^{gyro}=\left(\begin{array}{c} \frac{1}{4}I(2\dot{\theta }\dot{\rho }-\ddot{\psi }\sin\theta )\\ \frac{1}{4}I(\ddot{\theta }+\dot{\psi }\sin\theta (2\dot{\rho }+\dot{\psi }\cos\theta ))\\ \frac{1}{2}I(\ddot{\rho }+\ddot{\psi }\cos\theta -\dot{\theta }\dot{\psi }\sin\theta ) \end{array}\right)$$

This mathematical model was used to determine the output of the controller over the different actuation modalities implemented during the testing of the controller.

### 3.3. Controller Haptic Features

**Figure 3:**
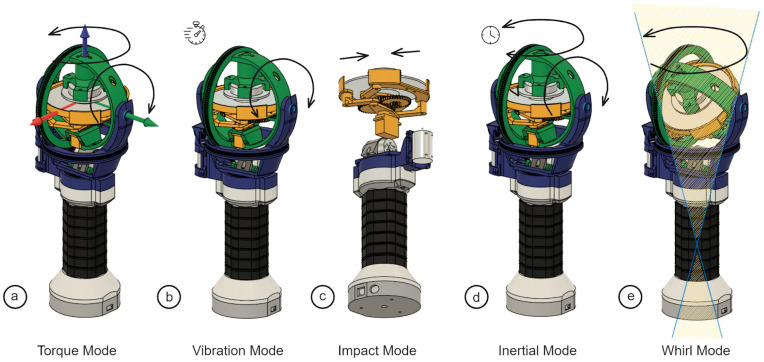
Operational Modes of the HapticWhirl Controller. (**a**) Torque Mode: Actuation of gimbals alters the flywheel’s orientation, generating torque. (**b**) Vibration Mode: Rapid pitch-axis oscillation of the flywheel produces tactile vibrations. (**c**) Impact Mode: Sudden halting of the flywheel simulates an impact. (**d**) Inertial Mode: Delayed flywheel response creates a sensation of drag. (**e**) Whirl Mode: Continuous yaw-axis actuation induces a stirring sensation.

During normal operation, the HapticWhirl controller is designed to function in transparent mode, where the flywheel is stabilized by the gimbals’ actuation. This stabilization ensures that the user does not feel any gyroscopic forces from the flywheel while moving the controller. However, the controller can manipulate its angular momentum to transition into five distinct haptic modes, each offering a unique interactive experience. These modes are shown in Figure 3 and described in this section.

In Torque Mode, as seen in Figure 3a, the controller actively produces feedback forces that the user can feel by manipulating the flywheel’s stabilized state through gimbal actuation. This actuation shifts the rotation plane of the flywheel, leveraging the law of conservation of angular momentum to generate the desired torque. While the device can operate transparently, not influencing the user’s movements, in Torque Mode it introduces a deliberate torque. This mode of haptic feedback, engaging the user with resistive or assisting forces, has been previously featured in prior works such as iTorque [23] and GyroVR [24].

In the Vibration Mode (Figure 3b), HapticWhirl employs a dynamic actuation of the gimbals to induce a rapid back-and-forth motion along the pitch axis. This action results in distinct low-frequency vibrations of higher magnitude than traditional vibrotactile actuators. When the flywheel is static, these induced vibrations are experienced as single-axis vector push-pull forces within the plane of the flywheel. Yet, when the flywheel is in rotation, the vibratory pattern becomes more complex due to the addition of a tangential component. This complexity emerges from the rotational movement, which overlays the basic push-pull oscillation with an additional directional force. increasing the magnitude of the vibration.

In Impact Mode (Figure 3c), the device uses its drum brake mechanism to suddenly halt the flywheel’s rotation, unlike the more gradual force feedback provided in Torque Mode. The sudden deceleration of the wheel almost instantaneously results in a high-magnitude torque that is felt by the user as a stark, jolting force. This immediate cessation of movement better replicates the abruptness of a real-world impact or collision than the torque mode, making it an ideal mode for replicating scenarios of hitting or crashing in virtual environments. Although this has been explored in other areas, such as self-assembly robotics [44], the use of a combination of gimbal actuation for kinesthetic feedback is a novel implementation.

Inertial Mode (Figure 3d) is achieved by introducing a latency into the HapticWhirl controller’s response, which alters the typical transparent interaction. This designed delay allows the gyroscopic forces of the flywheel to be partially felt by the user, emulating the sensation of inertia. As a result, when the user moves the device, they perceive a sense of resistance, as if they were maneuvering an object through a substance that opposes their motion. It replicates the feeling of pushing against a physical mass that resists immediate movement, providing a realistic sense of weight and drag. This intentional latency transforms the haptic experience to simulate various scenarios where inertia plays a significant role, such as navigating through viscous environments or handling heavy objects.

Lastly, the Whirl Effect (Figure 3e) represents a unique capability of the HapticWhirl controller to generate a sustained ‘stirring’ sensation or rotational forces. This is accomplished by maintaining the flywheel at a rotation angle and then actuating the yaw axis to spin continuously to change the orientation of the angular momentum. As the angular momentum ‘points’ in a new direction, a gyroscopic precession occurs, which the user feels as a sustained rotational force or a whirl effect. This can be particularly effective in simulating experiences that involve a continuous rotation, such as stirring a liquid or executing a spinning maneuver in a flight simulation.

Lastly, the Whirl Effect (Figure 3e) capitalizes on an innovative application of gyroscopic principles combined with the unique design of the HapticWhirl’s gimbal system, which permits unrestricted 360° rotation. This feature enables the flywheel to maintain a set rotational angle while the yaw axis is actuated to rotate without limits, altering the angular momentum’s orientation continuously. This action induces gyroscopic precession, where the rotational axis itself experiences rotation, producing a persistent ‘stirring’ force. Users experience this as a smooth, unending rotational motion, enhancing simulations that involve circular dynamics, such as mixing a substance or performing rotational maneuvers in aerial simulations. The device’s ability to simulate such sustained rotation without physical stops or resets by making use of the precession effect is a unique and novel approach proposed with the Whirl Mode.

## 4. Experimental Validation

In this section, we detail the experiments used to validate the forces produced by the HapticWhirl device.

### 4.1. Experimental Setup

Our experiment focused on validating the mathematical model and operational functionality of the HapticWhirl controller. For the setup, we used an ATI Mini40 force/torque sensor mounted on a handle grip to couple the controller. This allowed us to measure the force/torque exerted by the controller under various test conditions directly. A range of tests were then conducted, each designed to stimulate and measure the controller’s ability to generate the five haptic modalities: Torque, Vibration, Impact, Inertial and Whirl effects.

During the tests, telemetry data from the controller, including actual angular velocities, and gimbal orientations, were recorded for further analysis at 500 Hz. The objective of this experimental setup was to correlate these measurements with the predicted outcomes from the mathematical model (see [12] for more details on this), thereby validating the controller’s performance and the model’s accuracy.

### 4.2. Experimental Results

#### 4.2.1. Torque Feedback

To determine the maximum output torque of HapticWhirl, we programmed the flywheel to rotate at its maximum speed of 12,000 RPM while concurrently actuating the pitch gimbal at its peak speed of 800 RPM. To minimize potential external disturbances during the experiment, we secured the yaw axis (*Z*-axis) and only permitted rotation on the pitch axis (*Y*-axis). The flywheel’s orientation was actuated between −60 and 60 degrees, where the home position (0 degrees) corresponds to the disk rotating horizontally while the controller remains upright.

**Figure 4:**
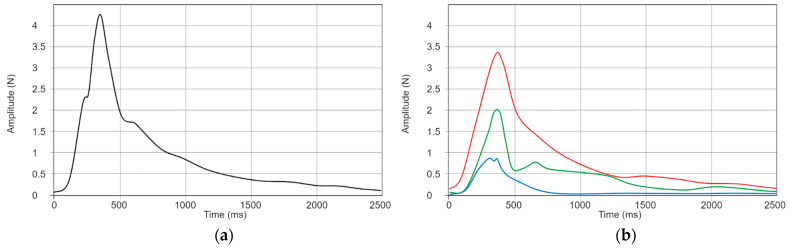
(**a**) Measurement of the magnitude of HapticWhirl’s output on a single axis. (**b**) A breakdown of forces for each axis—X (red), Y (green), and Z (blue).

We actuated the controller a total of ten times under these conditions, subsequently averaging the collected data prior to further calculations. The readings were processed through a Butterworth low-pass filter with a cut-off frequency of 50 Hz. Following this, we calculated the force magnitude using the formula:

(5) $$F_{magnitude}=\sqrt{F_{x}^{2}+F_{y}^{2}+F_{z}^{2}}$$

which delivered a peak total output of 4.34 N, as shown in Figure 4a. The output force felt by a user holding the controller will be perceived as a torque with two principal components, mainly exerted along the X and Y planes, as shown in Figure 4b.

#### 4.2.2. Impact Simulation

**Figure 5:**
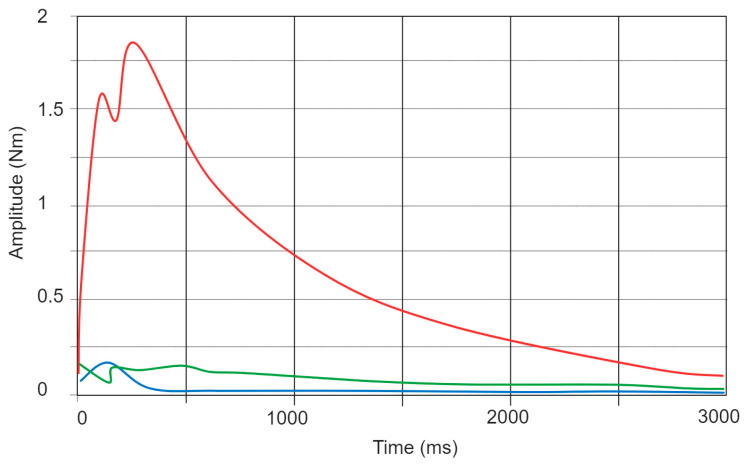
Output torque resulting from suddenly stopping the flywheel while it is rotating at full speed. Axes illustrated as follows: X (red), Y (green), and Z (blue).

In the impact simulation mode, the flywheel brake was used to create torque around the axis of rotation through a sudden stop of the flywheel. The drum brake could stop the flywheel in under 300 ms, which delivered a sudden peak of ~1.8 Nm, as shown in Figure 5. However, we have noted that such strong actuations limited the lifespan of the HapticWhirl, and so the next iteration of the HapticWhirl will include a redesign of the mechanism alongside a faster motor to combat this. Another advantage of using a faster motor and stronger mechanism is that it will allow us to use the brake to create different patterns of on/off braking. Due to the slow response of the current servo motor, we were limited to a maximum actuation of 10 Hz.

#### 4.2.3. Vibration Feedback

Oscillating the pitch axis around the home position at high speed generated back-and-forth vibrations. However, these oscillations are limited by the gimbal motors. In our testing, we ran this feature up to a frequency of 170 Hz with no actuation from the flywheel and up to 25 Hz with the flywheel rotating at maximum speed.

**Figure 6:**
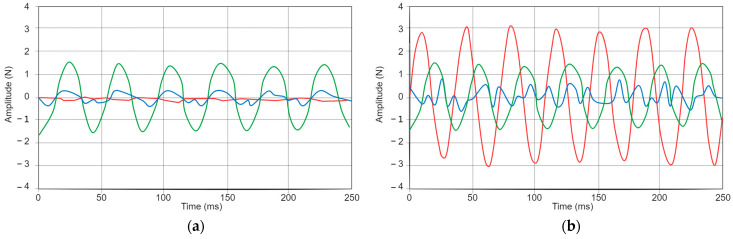
(**a**) Output from back-and-forth rotation of the flywheel gimbal (pitch) with the flywheel at rest. (**b**) Output the same rotation, but with the flywheel rotating at full speed. In both scenarios, the gimbal was actuated at a frequency of 25 Hz. Axes illustrated as follows: X (red), Y (green), and Z (blue).

The first approach had the advantage that it was easier to control and was capable of higher frequencies of vibration before the motors started to drift. The vibration generated has a clear directional property, aligned tangentially to the rotation axis, which provides clear directional cues. Figure 6a shows the measured output from this actuation. However, the feedback’s magnitude is comparatively lower than the second approach. In the second approach, activating the flywheel during the vibration pattern generation resulted in a higher magnitude output force. However, due to the limitations of the motors used to actuate the gimbals, when the flywheel was rotating at full speed, we could not achieve patterns higher than 25 Hz before the motors began to drift. Furthermore, in this scenario, the vibrations exhibited multi-component torque, as shown in Figure 6b.

#### 4.2.4. Whirl Effect (Rotational Forces)

**Figure 7:**
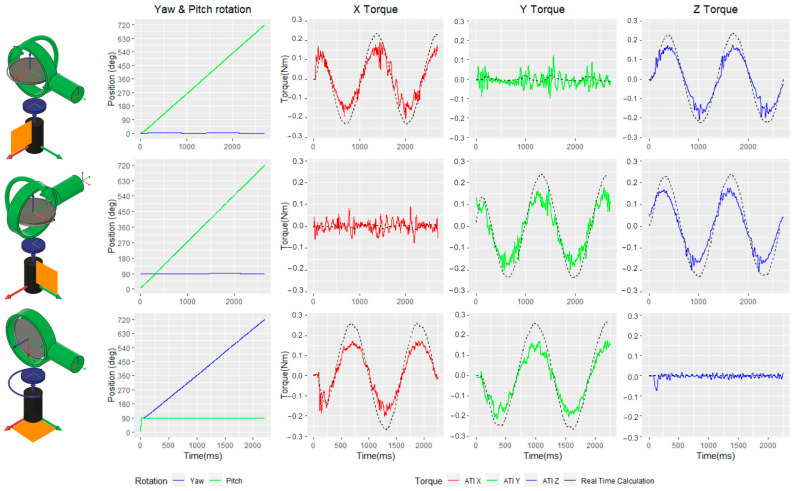
Measured outputs of the HapticWhirl when creating a whirl effect. The top row shows the rotation of the flywheel around its cente5 over the handle. The middle row displays the outputs and position when rotated with alignment to the handle’s *X*-axis. The bottom row illustrates the rotation of the flywheel around the handle’s *Z*-axis. In all instances, the active rotation axis was rotated at approximately 450 RPM. Axes illustrated as follows: X (red), Y (green), and Z (blue).

The output torque of our rotating flywheel is orthogonal to the rotation plane. Meaning when the controller remains upright and the disk is rotating horizontally, their Z-axes are aligned. However, if the disk is rotated in alignment to any other axis that is not the upward axis and spun around that axis, the flywheel will output a sinusoidal torque, as illustrated in Figure 3e. To illustrate this, we took measurements of the rotation of the flywheel around each of the handle axes. We observed that when the flywheel is aligned with the handle *Y*-axis and continuously rotates, there will be an output torque on the XZ plane. If the flywheel is aligned to the handle *X*-axis, the output torque will be along the YZ plane, and if the flywheel is rotated around the handle *Z*-axis, the output will be purely on the XY plane. To compile the relevant data, we took these measurements with gimbal rotations at 450 RPM with the disk aligned to each one of the handle axes. Under these conditions, we saw an almost consistent 0.2 Nm across each axis of the plane, while the axis to which the flywheel was aligned remained at zero, only registering noise. Figure 7 shows the measured torque and rotation values when producing this effect.

We used this test to also compare the mathematical model to the real-time output torque. This can be seen in the graphs as a black dotted line overlapping each axis in Figure 7. The estimated output torque aligns well with our readings, though it appears to offset the peaks consistently by ~0.05 Nm. This is to be expected, as the mathematical model does not account for friction or mechanical losses.

Finally, we should note that all the forces generated are well within the ranges that are perceivable by the hand-arm system and in line with those used in other systems and experiments, such as [23,45].

## 5. Applications

In virtual reality (VR) environments, interactions and collisions are typically experienced by the users as torque through a handheld controller or “proxy tool”. The HapticWhirl controller’s innovative design and features enable a wide array of force feedback simulations to enhance the user’s experience in such a scenario.

Firstly, the controller can generate torque through the manipulation of the flywheel and gimbal orientations. This unique characteristic enables the realistic replication of impact effects, such as the feedback experienced when holding a racket and striking a ball. This tangible force feedback amplifies the realism of interaction within a VR space and could be used in a variety of applications in gaming, training, and simulation.

Secondly, HapticWhirl can create distinctive vibration patterns by oscillating the gimbal. These pronounced vibrations, coupled with the ability to direct the forces along different axes, can simulate a diverse range of effects, including the shockwaves of an explosion or the recoil from firing a weapon with a decreasing magnitude in a VR game, as well as texture effects and vibrations from air and fluid flows in simulations or remote-control applications.

Moreover, the level of torque exerted by the flywheel is directly proportional to the gimbal axle’s rotation speed. This enables fine-grained control over haptic feedback, from simulating hard impacts to potential subtle guidance forces. Such nuanced manipulations can be employed to guide users through a VR space, adjust the perceived weight of virtual objects, or even correct a user’s hand position to align with the VR representation.

Finally, by utilizing the controller’s ability to continuously rotate on the yaw axis, we can generate a unique effect, a gyroscopic precession-like force. This feature is particularly useful to simulate the experience of stirring a fluid, for instance, where similar haptic forces are often encountered.

Overall, this wide range of feedback modalities allows HapticWhirl to offer a much greater range of opportunities for the design and creation of haptic effects than existing systems and thus provides a much greater scope for potential applications. Indeed, this increase in scope can allow designers to create new haptic experiences and, thus, potentially entirely new applications.

## 6. Discussion and Conclusions

In this paper, we introduce HapticWhirl, an innovative handheld haptic controller designed for virtual reality (VR) applications. The HapticWhirl stands out by exploiting the gyroscopic effects of a flywheel, utilizing it in diverse ways to create a range of haptic sensations. Unique to gyroscopic devices, HapticWhirl leverages gyroscopic precession, enabling torque generation without a fixed grounding point, and uses the flywheel’s rotational energy for dynamic torque output.

HapticWhirl is capable of delivering a force of 4.23 Newton at a distance of 160 mm from the anchor point, resulting in an approximate torque of 0.68 Nm. In comparison with previous gyroscopic devices, such as iTorqu [23] which generates a torque of 1.2 Nm, and the prototype developed by Nakamura et al. [25] with a peak torque of 1.1 Nm, our design demonstrates a lower peak torque. This discrepancy is partly attributed to our use of off-the-shelf components, unlike iTorqu’s custom-built flywheel, which is optimized for a higher moment of inertia and is over 30% heavier. Similarly, Nakamura’s design employs a flywheel that is twice as heavy. These figures are also lower than the capabilities of grounded devices like the Geomagic Touch [46], which exerts forces up to 3.3 N, and multi-actuator systems like Thor’s Hammer [5], capable of exerting 4 N in each of the six directions on three axes. The adequacy of HapticWhirl’s torque output and the potential for its enhancement will be the focus of future research, with user studies planned to assess the device’s effectiveness and explore possibilities for increasing its torque capabilities.

HapticWhirl is a handheld controller specifically designed to explore alternative control mechanisms of a flywheel actuator, aimed at generating a diverse range of kinaesthetic haptic modalities. HapticWhirl capitalizes on the gyroscopic effects of a flywheel, but its distinct gimbal design enables the flywheel to rotate a complete 360 degrees. Additionally, a brake has been incorporated to provide enhanced control over the flywheel’s movements.

The focus of this work lies in the exploration of novel operational methods that leverage gyroscopic principles. For instance, we explored a controlled delay in the transparency of the device to simulate inertia, offering a representation of weight and resistance in virtual environments, which will be further implemented in user studies in future work. Additionally, the implementation of a braking mechanism enables the simulation of sudden impacts and forces beyond the limitations of the gimbal actuation to maintain the orientation of the flywheel. Furthermore, HapticWhirl offers new possibilities with its capability for unlimited rotation in both gimbals, expanding the haptic interactions and capabilities to reorientate the disk and provide transparency operations that have not been previously explored in previous work.

Previous research has introduced various gyroscope-based haptic devices [22,24], yet the complex and often unintuitive nature of gyroscopic principles poses a significant challenge, particularly for multidisciplinary haptic researchers lacking extensive backgrounds in mathematical modeling. Recognizing this barrier, we have strived to demystify the operational mechanics of the HapticWhirl. We shared with this paper an online comprehensive, step-by-step guide to the mathematical modeling and operational mechanisms of gyroscopes used in the HapticWhirl [12]. Our aim is to bridge the gap between intricate gyroscopic theory and practical application, enabling a wider range of researchers to engage with and innovate in the field of haptic technology.

Future work will focus on improving the structural integrity of the device, specifically improving the axis of rotation for enhanced resilience under high-speed and high-torque operations. This refinement aims to balance the robustness of the device with a more compact and user-friendly form factor. The utilization of a faster motor and a smaller disk is anticipated to reduce the device’s overall size, thereby improving control and responsiveness.

Moreover, future work will focus on the user evaluation of the device and exploration of applications, as well as validating the five operational modes in VR environments. This exploration will include investigating potential retargeting applications that enable subtle repositioning of the user’s hand in VR without perceptible detection by gradually incrementing the torque as the user extends its arm.

In sharing our open-source design and detailed operation steps, our goal is to inspire further innovation and development in haptic technology. By providing a solid foundation and accessible resources, we aim to foster new haptic interactions and experiences, contributing to the evolution of immersive virtual environments.

## Data Availability

Data are contained within the article.
